# P2X7 receptor promotes the growth and metastasis of gastric cancer by activating P13/AKT/GSK-3 beta signaling (experimental research)

**DOI:** 10.1097/JS9.0000000000002406

**Published:** 2025-04-22

**Authors:** Wen-Jun Zhang, Hong-Liang Luo, Ji-Peng Liu, Yong-Sheng Xu, Wen-Long Wang, Chao Huang

**Affiliations:** a Department of Rehabilitation Medicine, The second affiliated hospital, Jiangxi Medical College, Nanchang University, Nanchang city, Jiangxi province, China; b Department of Gastrointestinal Surgery, The second affiliated hospital, Jiangxi Medical College, Nanchang University, Nanchang city, Jiangxi province, China; c Department of Emergency Medical, The second affiliated hospital, Jiangxi Medical College, Nanchang University, Nanchang city, Jiangxi province, China

**Keywords:** ATP, GC, metastasis, P2X7R, proliferation, treatment

## Abstract

**Objective:**

This study investigated the role of P2X7 receptor (P2X7R) in the proliferation and metastasis of gastric cancer (GC).

**Methods:**

The functional role and possible mechanism of P2X7R in the progression of GC were investigated through *in vitro* and *in vivo* experiments.

**Results:**

The results showed that ATP and its analogue BzATP increased calcium current in AGS and HGC-27 cells, while P2X7R antagonists A438079 and AZD9056 decreased the ATP-induced calcium influx. Activation of P2X7R increased the glycogen accumulation in GC cells, enhanced the stress ability of actin fibers and cell morphology changes, and promoted the proliferation, migration and invasion of GC cells. Conversely, the application of A438079, AZD9056 or siP2X7R inhibited the proliferation, migration and invasion of GC cells. Moreover, activation of P2X7R increased the expression levels of EMT/metastasis related genes MMP-2, MMP-9, N-cadherin, Zeb1, Vimentin and Snail, hut decreased the E-cadherin expression. While A438069, AZD9056, LY294002 or siP2X7R reversed the expression of the above genes. Activation of P2X7R activated P13/AKT/GSK-3beta signaling to promote the proliferation, migration and invasion of GC. Additionally, *in vivo* experiments showed that ATP activated P2X7R to induce the growth of tumors.

**Conclusions:**

Our conclusion is that activation of P2X7R promotes the proliferation, metastasis and EMT of GC cells by activating P13/AKT/GSK-3beta signaling, and indicates that P2X7R may become a new potential target for GC treatment.

## Introduction

The pathological mechanism of gastric cancer (GC) is complex and involves many molecular bases^[1,2]^. Therefore, exploring the molecular basis related to the pathological mechanism of GC and finding new therapeutic targets has become an urgent issue in the treatment of GC.

It has been found that ATP has a certain effect on tumor progression^[^3^]^. ATP can interact with other cells or molecular substances in the tumor microenvironment (such as P2X purinergic receptors and immune cells), and indirectly regulates the progression of tumors^[^4,5^]^. P2X7R is dependent on ATP ion channel receptors and plays a key role in the response to many diseases^[^6^]^. Recently, it has been discovered that the P2X7R plays an important regulatory role in tumor progression, which has attracted the attention of researchers^[^7^]^. The tumor microenvironment contains a large amount of ATP, which can be released by tumor cells or other cells and serves as a natural activator of P2X7R. The activated P2X7R can open the cation channel on the cell membrane (mainly calcium ion influx), activate intracellular signaling (such as AKT), and play an important regulatory role in the growth, proliferation, apoptosis, migration and metastasis of tumor cells^[^5,8^]^. The study found that the high expression of P2X7R promoted the metastasis of colon cancer cells, and found that P2X7R could be used as an independent indicator of the prognosis of colon cancer^[^9^]^. Studies have found that ATP-induced activation of P2X7R activates the AKT pathway and promotes the migration and metastasis of human breast cancer cells^[^10^]^. The expression level of P2X7R in breast cancer tissue was higher than that in normal breast tissue. Increased expression of P2X7R promoted the proliferation of breast cancer cells, while knockdown of P2X7R expression by P2X7R-shRNA inhibited the proliferation of breast cancer cells^[^11^]^.

**HIGHLIGHTS**

1. The expression pattern of P2X7 receptor in gastric cancer was investigated.
2. P2X7 receptor activation can promote the progression of gastric cancer by activating P13/AKT/GSK-3 beta signaling.
3. Antagonism of P2X7 receptor activity has pharmacological effects on inhibiting the growth of gastric cancer.
4. P2X7 receptor activation promotes tumor growth in vivo.

These studies indicate that there is a close relationship between P2X7R and tumors. However, the role of P2X7R regulating the progression of GC has been poorly understood. Therefore, it is of great significance to investigate the role of P2X7R in GC. In this study, we investigated the potential role of P2X7R regulating the proliferation and metastasis of GC, providing a new theoretical basis and data support for the treatment of GC in the future.

## Methods and materials

### Cell culture

AGS and HGC-27 cell lines (Cell Bank of the Chinese Academy of Sciences, Shanghai, China) were cultured with 1640 medium containing 10% FBS. The petri dish was cultured in an incubator and the cell culture medium was changed every 3 days.

### Protein quantitative analysis

Total protein was extracted from AGS and HGC-27 cells. According to the molecular weight indicated by the marker, cut out the target bands and the internal bands. The strips were placed into the rabbit P2X7R primary antibody (1:500, Millipore, Bedford, MA, USA), rabbit E-cadherin primary antibody (1:1000, China Wuhan Bosher Co., Ltd), rabbit vimentin primary antibody (1:1000, China Wuhan Bosher Co., Ltd), rabbit snail primary antibody {1:1000, Sanying Biotechnology Co., Ltd. Wuhan, China), rabbit N-cadherin primary antibody (1:1000, China Wuhan Bosher Co., Ltd), or rabbit β-actin antibodies (1:1000, China Wuhan Bosher Co., Ltd) for overnight at 4℃. Next day, the strips were washed with 1xTBST, then incubated in goat anti-rabbit secondary antibody (1:5000, Wuhan Boshide Co., Ltd) for 1 h, subsequently, the bands were subjected to chemical immune reaction and data analysis.

### Cell scratch assay

GC cells were inoculated (approximately 7 × 10^5^ cells) in the 6-well plate, waited for the cells to be covered. Used 10 μl gun head for vertical scratching and washed the scratched cells with PBS, observed and photographed under an inverted microscope at 0 h and 24 h, respectively, and wound healing rate between cell scratches was calculated.

### Cell migration and invasion assays

The chamber was smeared with Matrigel before use (omitted from the migration assay), 200 µl serum-free GC cell suspension (2x10^4^ cells) was planted, 200ul 1640 culture medium containing 10%FBS was added to the 24-well plate, and then the chamber was placed in the 24-well plate. 400 μl methyl violet was added to stain the migrating or invasive cells in the chamber, and then observed and photographed under an inverted microscope.

### Fluo-3AM assay

The intracellular calcium concentration of GC cells was measured by using fluorescence indicator Fluo-3AM (Yuheng Biological Technology Co., Ltd. Suzhou, China). GC cells were inoculated in 24-well plates and were washed with PBS, and 300 µl 4 µM Fluo-3AM working solution was added for 30 min. Then, added 400 µl PBS and incubated for 10 min, fluo-3 fluorescence signals were detected by using a laser confocal microscope (Danmic Global, LLC, San Jose, CA, USA).

### Actin labeling assay

GC cells were inoculated in 24-well plates, then were washed by PBS. The 24-hole plate was placed on the ice and fixed with 4% paraformaldehyde for 30 min, and washed with PBS. Added 200 μl 0.5% Trion X-100 to react for 10 min, and washed with PBS. Then, 200 μl of PBS diluted 3 μl of YF fluorescently labeled phalloidin solution was added for 30 min, and washed with PBS. Finally, the changes of cell morphology and actin fibers were observed under inverted fluorescence microscope.

### PAS glycogen content detection

AGS and HGC-27 cells were seeded in a 24-well plate, PAS fixative was added for 20 min and then washed three times with PBS. Oxidant was added for 15 min. The cells were washed with sodium sulfite solution twice, and then washed with PBS. Mayer hematoxylin dye solution was added for 2 min, then washed with PBS. Then, the number of PAS-positive cells was observed under an inverted microscope and counted using Image Pro Plus 6.0.

### EDU assay

AGS and HGC-27 cells were inoculated onto 24-well plates, 100 μl EDU working solution was added and incubated for 2 h. Cells were fixed with 4% paraformaldehyde for 15 min. Then 50 μl 2 mg/ml glycine solution was added to react for 5 min, and 0.3%Trix-100 PBS was added to react for 10 min. Then, Apollo and Hoechst staining solution were added for staining.

### Small interference RNA transfection

P2X7R siRNA (siP2X7) (5-CCGAGAAACAGGCGAUAAU-3) and a scramble sequence not targeting any known gene was used as a control siRNA (siCon) (Boshide, Wuhan, China). GC cells were seeded in 24-well plates at a density of 1 × 10^5^/ml. After 6 h, the cells were transfected with siP2X7 or siCon by using Lipofectamine 3000. After 40 h of transfection, Western-blotting and qRT-PCR were used to detect the gene knockout efficiency, and three repeated experiments were carried out to verify the gene knockout effect.

### In vivo experiment

The work has been reported in accordance with the ARRIVE guidelines (Animals in Research: Reporting In Vivo Experiments)^[^12^]^. 18 BALB/c nude mice were reared in an aseptic environment controlled by light and temperature in the laboratory. AGS cells were collected and reconstituted in PBS (100 μl), and approximately 4 × 10^6^ cells. Cells were injected subcutaneously on the flank regions of 4-week-old male nude mice. When the diameter of the tumor was close to 5 mm, 18 BALB/c nude mice were randomly divided into 3 groups, with 6 mice in each group. The PBS (control), ATP (200 µM), or ATP + AZD9056 (10 µM) were injected to xenotransplant tissue at twice a week. The tumor size was measured with Vernier calipers, induced tumor volume = [length x width^2^]/2 for about 1 month.

### Statistical method

Data was presented as mean ± SEM by using GraphPad program software. Differences between the two groups were assessed using Student t test. Differences between three or more groups were assessed by one-way analysis of variance followed by Tukey’s post-hoc test. *P* < 0.05 is considered to be statistically significant.

## Results

### Expression of P2X7R in GC cells

**Figure 1.**
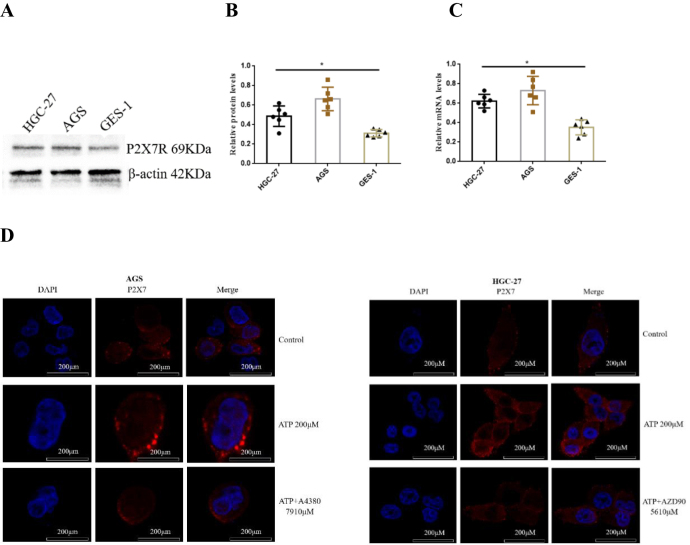
Expression of P2X7R in GC cell lines and GES-1 cell line. **(A-C)** Western-blotting and qRT-PCR were used to detect the expression of P2X7R in GC cells. **(D)** AGS and HGC-27 cells were treated with ATP (200 μM), ATP + A438079 (10 μM) and ATP + AZD9056 (10 μM). The expression and localization of P2X7R in GC cells were detected by immunofluorescence. Data are expressed as the mean ± SEM. Student’s t-test or one-way ANOVA followed by Tukey’s *post hoc.* (n = 6). * *P* < 0.05.

To investigate whether P2X7R is expressed in GC cells. Western-blotting and qRT-PCR were used to detect the expression of P2X7R protein and mRNA in GC cells (AGS and HGC-27) and normal gastric mucosal epithelial cell line (GES-1). The results showed that P2X7R was expressed in GC cells, which was higher than that in GES-1 cells (Fig. 1A-1C). In addition, the expression of P2X7R in GC cells was further identified by immunofluorescence. The results showed that P2X7R was expressed on the membrane of GC cells, and ATP enhanced the fluorescence intensity of P2X7R labeled cells. While the application of P2X7R antagonists A438079 and AZD9056 reduced the increase of ATP-induced fluorescence intensity (Fig. 1D). These data suggest that P2X7R may play a role in the progression of GC cells.

### Activation of P2X7R increases calcium influx in GC cells

**Figure 2.**
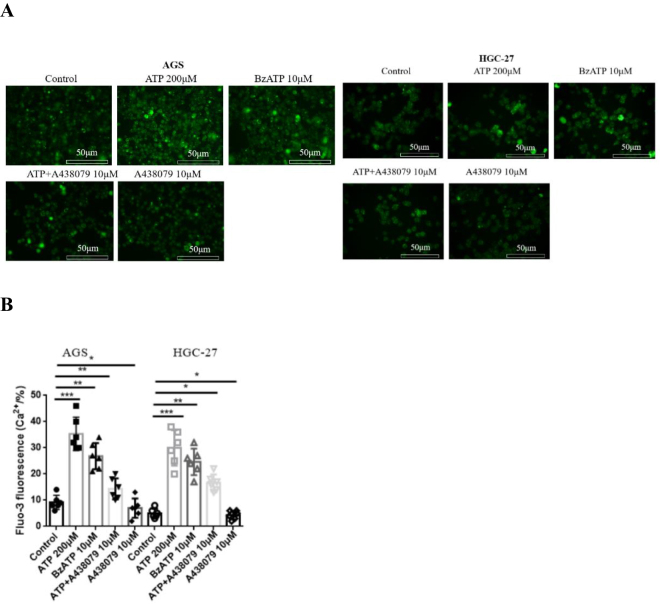
Effect of P2X7R on calcium influx in GC cells. Fluo-3AM was used to detect the changes of intracellular calcium concentration in GC cells. Data are expressed as the mean ± SEM. Student’s t-test or one-way ANOVA followed by Tukey’s *post hoc.* (n = 6). * *P* < 0.05, **P < 0.01, ***P < 0.001.

P2X7R activation can open cation channels (mainly calcium influx) on the cell membrane and regulate the life activity of tumor cells^[^13,14^]^. Therefore, based on the expression of P2X7R in GC cells, Flu-3AM calcium fluorescence probe was used to detect calcium concentration in GC cells. AGS and HGC-27 cells were treated with 30 min by ATP (200 μM), BzATP (10 μM), A438079 (10 μM) and ATP + A438079 (10 μM). The results showed that ATP and BzATP significantly increased the intracellular calcium current in GC cells. However, the use of P2X7R inhibitor A438079 reduced the increase of calcium current induced by ATP. Interestingly, in the absence of ATP, A438079 alone reduced the basic intracellular calcium concentration (Fig. 2A-2B). The possible reason is that there is a certain active state of P2X7R under the basic conditions. These data suggest that P2X7R plays a certain functional role in GC cells.

### Activation of P2X7R promotes the proliferation of GC cells

**Figure 3.**
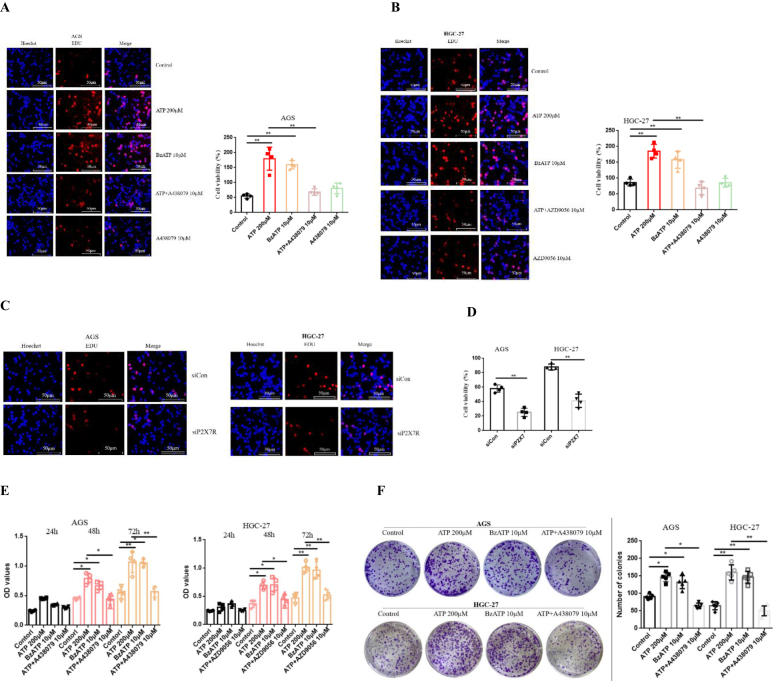
Effect of P2X7R on the proliferation of GC cells. AGS and HGC-27 cells were treated with ATP (200 μM), BzATP (10 μM), A438079 (10 μM), AZD9056 (10 μM), ATP + A438079 (10 μM) or ATP + AZD9056 (10 μM) or siCon/siP2X7R. **(A-D**): EDU was used to detect the proliferation ability of GC cells. **(E**): the ability of cell proliferation was detected by CCK-8. **(F**): the clone forming ability of GC cells was detected by plate cloning test. Data are expressed as the mean ± SEM. Student’s t-test or one-way ANOVA followed by Tukey’s *post hoc*. (n = 4). * *P* < 0.05, **P < 0.01, ***P < 0.001.

AGS and HGC-27 cells were treated with ATP (200 μM), BzATP (10 μM), A438079 (10 μM), AZD9056 (10 μM), ATP + A438079 (10 μM) or ATP + AZD9056 (10 μM) for 24 h. Detection of proliferation ability of GC cells by EDU. As expected, ATP and BzATP promoted the proliferation of GC cells, while A438079 and AZD9056 inhibited the proliferation of GC cells induced by ATP. However, in the absence of ATP, the inhibitory effect of P2X7R antagonist on cell activity was not obvious (Fig. 3A-3B).

Moreover, in order to further verify the above results, siRNA was used to transfect AGS and HGC-27 cells to knock down the expression of P2X7R, and Western-blotting and qRT-PCR were used to determine the knockdown efficiency. The results showed that knocking down the expression of P2X7R in GC cells inhibited the proliferation of GC cells (Fig. 3C-3D). Moreover, CCK-8 assay showed similar results for cell proliferation (Fig. 3E). In addition, Fig. 2F showed that ATP and BzATP increased the ability of clone formation of GC cells, while A438079 inhibited the increase of clone formation induced by ATP. These data show that the activation of P2X7R promotes the proliferation of GC cells.

### Activation of P2X7R promotes migration and invasion of GC cells

**Figure 4.**
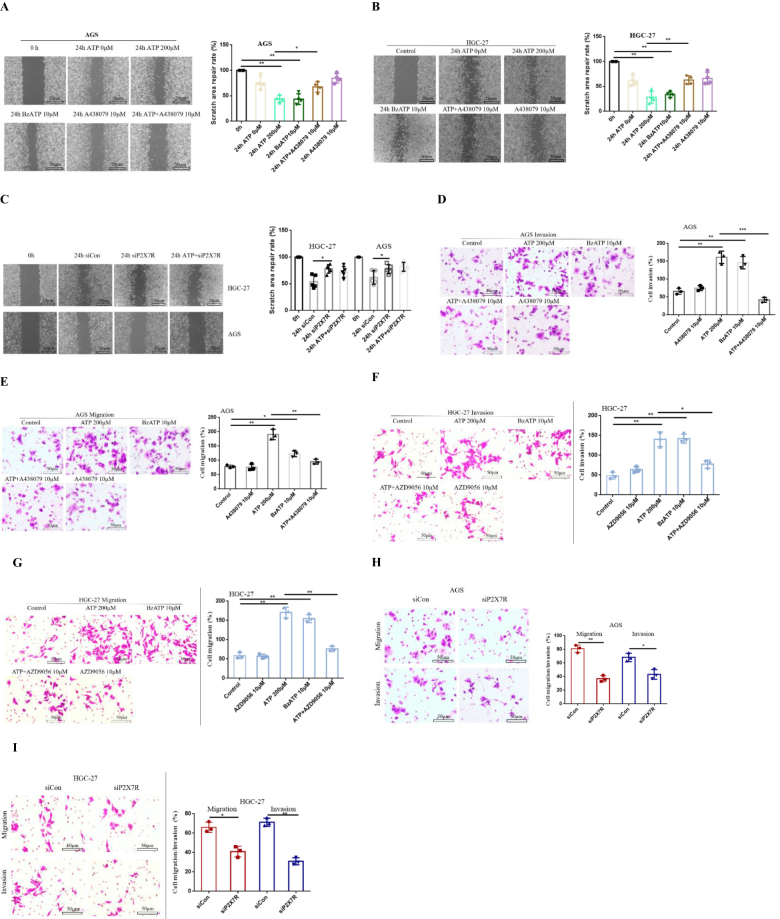
Effect of P2X7R on migration and invasion of GC cells. Cells were treated or untreated with ATP (200 μM), BzATP (10 μM), A438079 (10 μM), ATP + A438079 (10 μM), AZD9056 (10 μM), ATP + AZD9056 (10 μM) or siCon/siP2X7 for 24 h. **(A-C**): The migration ability of P2X7R to GC cells was detected by cell scratch test. **(D-I**): Transwell migration and invasion assay was used to detect the migration and invasion ability of GC cells. Data are expressed as the mean ± SEM. Student’s t-test or one-way ANOVA followed by Tukey’s *post hoc*. (n = 3-4). * *P* < 0.05, **P < 0.01.

To investigate whether the activation of P2X7R can affect the migration and invasion of GC cells. AGS and HGC-27 cells were treated with ATP (200 μM), BzATP (10 μM), A438079 (10 μM), ATP + A438079 (10 μM), AZD9056 (10 μM), ATP + AZD9056 (10 μM) or siP2X7R/siCon for 24 h. The results showed that ATP and BzATP promoted the migration of GC cells, while A438079 inhibited the migration ability of GC cells induced by ATP. However, in the absence of ATP, the use of antagonists had no significant inhibitory effect on the migration of GC cells (Fig. 4A-4B). In addition, the expression of P2X7R in GC cells was knocked down by siRNA transfection, and the knockdown efficiency was verified by Western-blotting and qRT-PCR. The results showed that knockdown of P2X7R expression decreased the migration ability of GC cells. However, on the basis of siP2X7R treatment, no obvious migration promotion effect was found by using ATP (Fig. 4C).

Moreover, Transwell invasion and migration assay was used to detect the invasion and migration ability of GC cells. Figure 4D-4G showed that ATP and BzATP promoted the invasion and migration of GC cells, while A438079 and AZD9056 inhibited the increase of invasion and migration induced by ATP. However, the inhibitory effect of P2X7R antagonist on the migration and invasion of GC cells was not significant. In addition, the above results were verified using siRNA to knock down P2X7R expression in GC cells. The P2X7R knockdown efficiency was determined by qRT-PCR and Western-blotting. Similar results were obtained after siRNA knocked down P2X7R expression (Fig. 4H-4I). These data confirm that activation of P2X7R enhances the migration and invasion ability of GC cells.

### Activation of P2X7R enhances the stress response and morphological changes of actin fibers in GC cells

**Figure 5.**
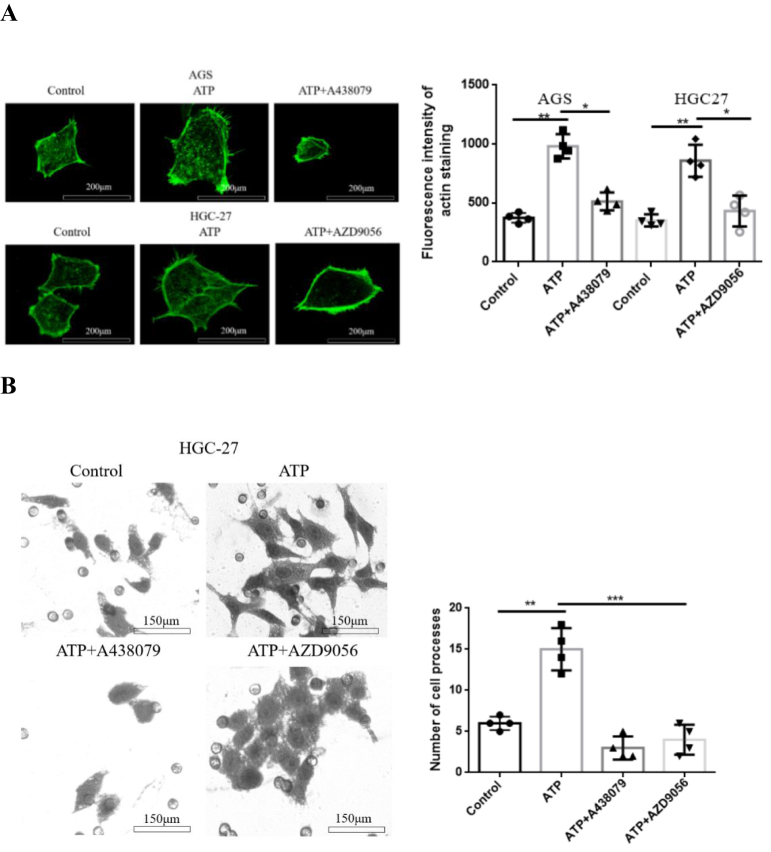
Effect of P2X7R on actin and cell morphology of GC cells. **(A**): YF- actin assay detected the changes of actin in AGS and HGC-27 cells. **(B**): methyl violet staining was used to detect the morphological changes of HGC-27 cells induced by P2X7R activation and inhibition. Data are expressed as the mean ± SEM. Student’s t-test or one-way ANOVA followed by Tukey’s *post hoc*. (n = 4). ** *P* < 0.01, ***P < 0.001.

Actin filaments in cells play a key role in cell movement and migration, and the enhancement of actin fibers can accelerate the movement ability of cells^[^15,16^]^. Therefore, in order to understand the possible mechanism of P2X7R regulating GC cell motility. YF actin staining test was used to detect the changes of actin filaments in GC cells. AGS and HGC-27 cells were treated with ATP (200 μM), ATP + A438079 (10 μM) and ATP + AZD9056 (10 μM) for 24 h. Compared with the control group, the actin fibers in the ATP group became thicker and longer, more pseudopodia could be seen at the edge of the cells, the cell size increased and showed fusiform changes, which enhanced the stress response of actin filaments in GC cells. However, after treatment with P2X7R antagonist A438079 or AZD9056, the actin filaments were significantly reduced, shortened, the cells showed oval changes, the pseudopod protruding from the cells became sparse, and the stress changes of actin filaments induced by ATP was weakened (Fig. 5A).

In addition, in order to understand the effects of P2X7R activation and inhibition on the morphology of GC cells. Through the above treatment, the morphological changes of HGC-27 cells were observed by methyl violet staining on the cells passing through matrix glue. Compared with the control group, the cells in the ATP treatment group showed long fusiform changes and increased in volume. It was found that the connection between the cells was closer. On the basis of ATP treatment, A438079 and AZD9056 antagonist treatment found that the cells showed round-like changes, filamentous processes became smaller or even disappeared, and the connection between cells decreased, weakened the changes of cell morphology (Fig. 5B). These data suggest that P2X7R activation promotes the motility and migration of GC cells.

### Activation of P2X7R increases glycogen accumulation in GC cells

**Figure 6.**
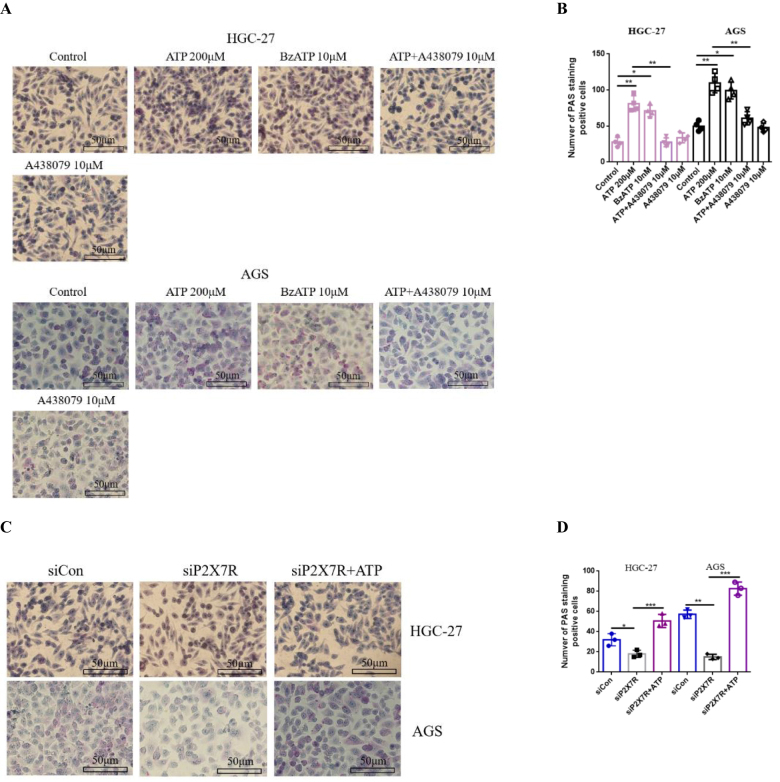
Effect of P2X7R on glycogen content in GC cells. AGS and HGC-27 cells were treated with ATP (200 μM), BzATP (10 μM), ATP + A438079 (10 μM), siCon/siP2X7R or siPX7R + ATP for 24 h. PAS glycogen staining was used to detect the changes of glycogen content in GC cells. Data are expressed as the mean ± SEM. Student’s t-test or one-way ANOVA followed by Tukey’s *post hoc*. (n = 3-4). * *P* < 0.05, **P < 0.01, ***P < 0.001.

To investigate whether P2X7R can affect the metabolism of glycogen in GC cells. AGS and HGC-27 cells were treated with ATP (200 μM), BzATP (10 μM), A438079 (10 μM) or ATP + A438079 (10 μM) for 24 h. The results of PAS staining showed that dots and small blocks of red (changes after glycogen or polysaccharide staining) could be seen in the cytoplasm of GC cells, especially in AGS cells. ATP and BzATP increased glycogen accumulation in GC cells. Interestingly, P2X7R antagonist A438079 reduced ATP-induced glycogen accumulation in GC cells. However, in the absence of ATP, the effect of intracellular glycogen alone on A438079 was not significant (Fig. 6A-6B).

Next, siRNA was used to transfect GC cells to knock down the expression of P2X7R, Western-blotting and qRT-PCR were used to determine the knockdown efficiency, and the change of intracellular glycogen content was detected. The results showed that compared with siCon treatment group, siP2X7R decreased the glycogen content of GC cells. However, it is interesting to find that on the basis of siP2X7R treatment, the use of P2X7R agonist ATP increased the accumulation of intracellular glycogen content (Fig. 6C-6D). These data reveal that the activation of P2X7R enhances the activity and glycogen metabolism of GC cells.

### P2X7R promotes the growth, migration and invasion of GC cells by activating P13/AKT/GSK-3beta signal

P13/AKT is a key signal pathway in cells and plays an important regulatory role in tumor progression^[^17^]^. AKT/GSK-3β activation promotes tumor growth, metastasis, and EMT formation (the expression of EMT markers such as Vimentin, and Snail)^[^18,19^]^. However, blocking AKT phosphorylation can down-regulate the expression of downstream GSK-3β and inhibit the expression of EMT-related genes such as Vimentin, Snail and β-catenin, thereby inhibiting tumor metastasis^[^20,21^]^. These studies have revealed that P13/AKT/GSK-3β is a key regulatory pathway in tumor progression. However, it is not clear whether P2X7R can mediate this signal to regulate the proliferation and metastasis of GC cells. Therefore, we further investigated whether P2X7R can activate this signal on GC. AGS and HGC-27 cells were treated with ATP (200 μM), BzATP (10 μM) and ATP + A438079 (10 μM). The protein expressions of p-Akt and p-GSK-3beta were detected by Western-blotting. Figure 7A and 7B showed that ATP and BzATP increased the protein expression of p-Akt and p-GSK-3beta, while A438079 decreased the increase of p-Akt and p-GSK-3beta protein expression induced by ATP. In addition, in order to verify the above experimental results, siRNA was used to transfect AGS and HGC-27 cells to knock down the expression of P2X7R. It was found that knocking down the expression of P2X7R decreased the expression of p-Akt and p-GSK-3beta protein in GC cells (Fig. 7C).

**Figure 7.**
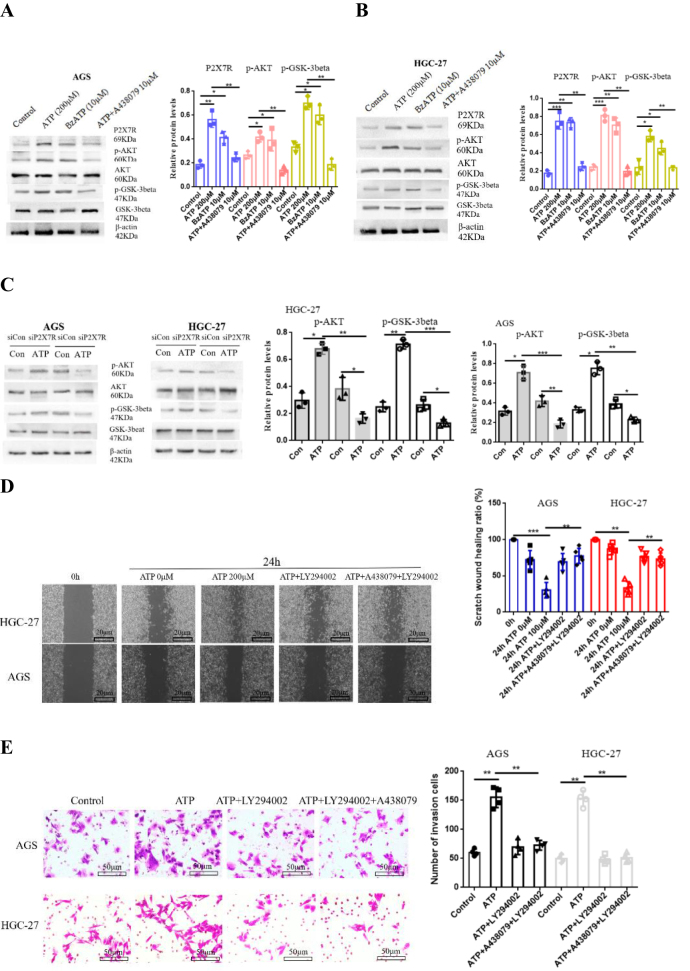
P2X7R mediates the effect of P13/AKT/GSK-3beta signal on GC cells. **(A and B**): The protein expression of p-AKT and p-GSK-3beta were detected by Western-blotting. **(C**): siRNA was transfected into AGS and HGC-27 cells, and the protein expression of p-AKT and p-GSK-3beta was detected by Western-blotting. **(D-G**): Cell scratch, Transwell invasion, PAS staining and CCK-8 were used to detect the proliferation, migration and invasion of GC cells. Data are expressed as the mean ± SEM. Student’s t-test or one-way ANOVA followed by Tukey’s *post hoc*. (n = 3-5). * *P* < 0.05, **P < 0.01, ***P < 0.001.

**Figure 7b.**
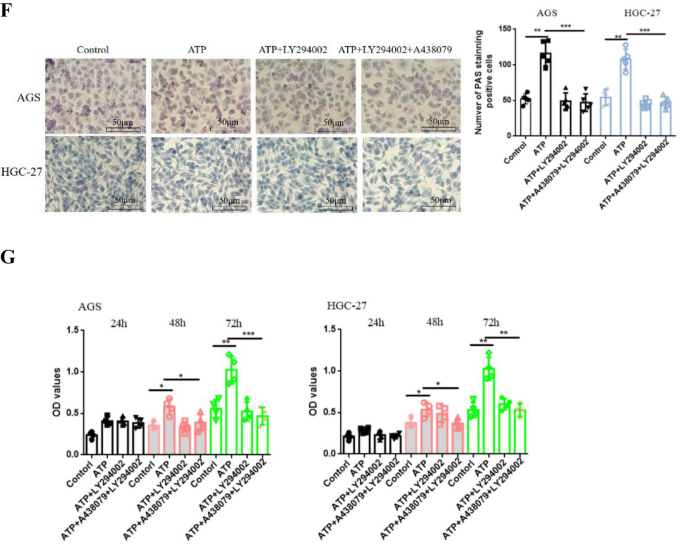

On the premise of obtaining the above experimental results, in order to better understand the effect of P13/AKT/GSK-3beta signal on GC cells, the participation of P2X7R is needed. AGS and HGC-27 cells were treated with LY294002 (10 mM), an inhibitor of this pathway. The effects of P13/AKT/GSK-3beta signaling pathway activation on GC cell activity were investigated by cell scratch, Transwell invasion, PAS glycogen staining and CCK-8 assay. The results showed that LY294002 inhibited the proliferation, migration and invasion of GC cells induced by ATP. However, no significant synergistic effect was found in the combination of A438079 and LY294002 (Fig. 7D-7G). These data suggest that P2X7R activation may promote the growth, migration and invasion of GC cells by regulating P13/AKT/GSK-3beta signal.

### P2X7R regulates the expression of EMT/ metastasis related genes

**Figure 8.**
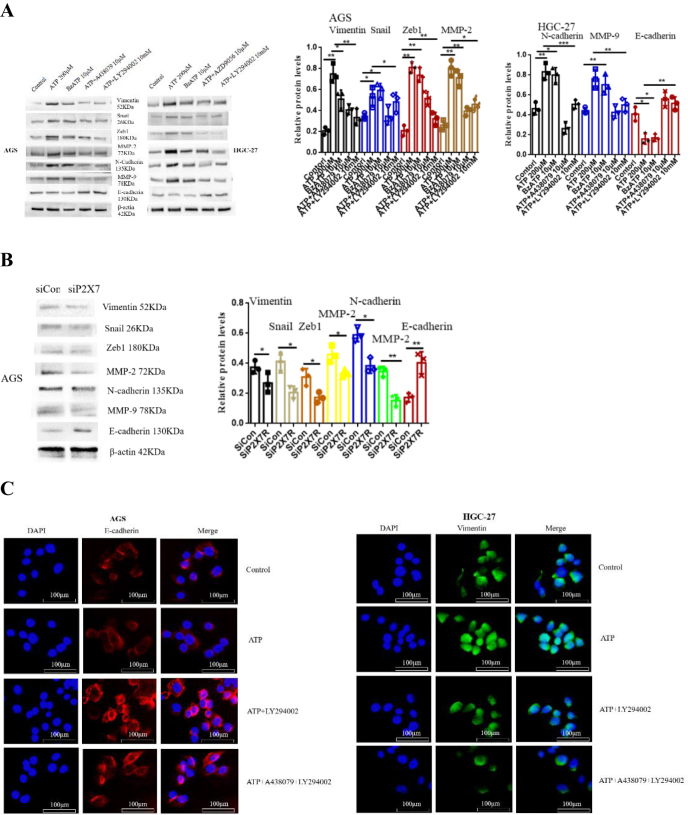
P2X7R regulates the expression of EMT/ metastasis related genes. **(A**): ATP (200 μM), ATP + A438079 (10 μM), ATP + AZD9056 (10 μM) and ATP + LY294002 (10 mM). The expression of MMP-2, MMP-9, N-cadherin, Zeb1, Vimentin, Snail and E-cadherin was detected by Western-blotting. **(B**): AGS cells were treated with siCon/siP2X7R, and the protein expression of the above related genes was detected by Western-blotting. **(C**): immunofluorescence to detect the expression of Vimentin and E-cadherin in GC cells. Data are expressed as the mean ± SEM. Student’s t-test or one-way ANOVA followed by Tukey’s *post hoc*. (n = 3). * *P* < 0.05, **P < 0.01.

EMT is an important feature of tumor metastasis^[^22,23^]^. Therefore, this study examined the effect of P2X7R on the expression of EMT/ metastasis related genes. Western-blotting was used to detect the protein expression of EMT/ metastasis related markers MMP-2, MMP-9, N-cadherin, Zeb1, Vimentin, Snail and E-cadherin in gastric cancer cells. AGS and HGC-27 cells were treated with ATP (200 μM), BzATP (10 μM), ATP + A438079 (10 μM), ATP + AZD9056 (10 μM) and ATP + LY294002 (10 mM) for 24 h. Figure 8A showed that ATP and BzATP increased the expression of MMP-2, MMP-9, N-cadherin, Zeb1, Vimentin and Snail, while inhibited the expression of E-cadherin. However, A438079, AZD9056 and LY294002 could reverse ATP-induced changes in the expression of these genes. Moreover, siRNA knocked down the expression level of P2X7R in AGS cells, and the knockdown efficiency was verified by Western-blotting, and similar results were obtained (Fig. 8B).

In addition, the expression of Vimentin and E-cadherin in the above genes was detected by immunofluorescence. The results showed that ATP enhanced the fluorescence intensity of Vimentin and decreased the fluorescence intensity of E-cadherin. On the contrary, P2X7R antagonist and LY294002 attenuated ATP-induced changes in fluorescence intensity of Vimentin and E-cadherin (Fig. 8C). These data suggest that P2X7R may have a regulatory effect on EMT formation in GC.

### Extracellular ATP promotes tumor growth in vivo

**Figure 9.**
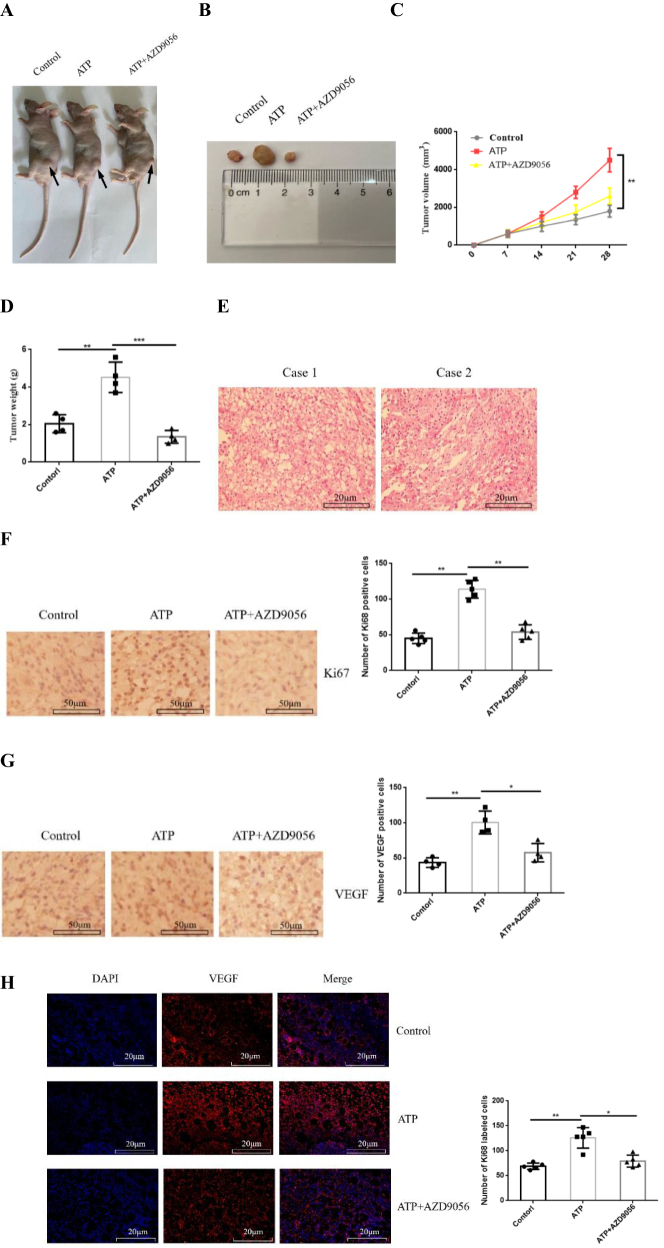
Effect of extracellular ATP activating P2X7R on tumor growth *in vivo*. **(A-D**): P2X7R activation promotes tumor growth in nude mice (Subcutaneous tumor growth indicated by black arrow). (E): HE staining was used to detect the pathological changes of tumor formation in nude mice. (F and G): Immunohistochemical staining was used to detect the expression of Ki67 and VEGF in tumor tissues. (H): Immunofluorescence was used to detect the expression of VEGF in tumor tissues. Data are expressed as the mean ± SEM. Student’s t-test or one-way ANOVA followed by Tukey’s *post hoc*. (n = 3-4). * *P* < 0.05. **P < 0.01. ***P < 0.001.

The effect of P2X7R activation on the growth of GC was observed *in vivo*. 18 BALB/c nude mice were randomly divided into 3 groups, 6 mice in each group: PBS group (control group), ATP (200 μM) group and ATP + AZD9056 (10 μM) group. The volume of induced tumor was measured every week. The results showed that: compared with the control group, ATP treatment group significantly induced tumor growth and increased the volume and weight of tumors. AZD9056 inhibited ATP-induced tumor growth (Fig. 9A-9C).

In addition, on day 28, tumor tissues were taken out and H-E staining showed the pathological changes of tumorigenesis in nude mice (Fig. 7E). Moreover, Ki67 and VEGF are commonly used markers for tumor growth, and their high expression are closely related to tumor growth^[^24,25^]^. However, whether inhibiting P2X7R activity can reduce the expression of Ki67 and VEGF in GC tissues *in vivo*. Therefore, the expression of Ki67 and VEGF in tumor tissues of each group were detected by immunohistochemistry and immunofluorescence. Compared with the control group, the number of Ki67-and VEGF-labeled positive cells in tumor tissues was significantly increased in ATP-treated group, while the expression of Ki67-and VEGF-labeled positive cells in tumor tissues was reduced after ATP + AZD9056 treatment (Fig. 7F-7H). These data suggest that P2X7R activation promotes the growth of GC cells *in vivo*.

## Discussion

GC treatment has always been a difficult problem currently facing^[^26^]^. Therefore, in-depth exploration and mining of the relevant molecular basis involved in the pathogenesis of GC and finding effective anti-cancer targets have become an urgent problem to be solved. Currently, the role of P2X7R in regulating tumor progression has received widespread attention. Studies have shown that P2X7R is highly expressed in the tissues of patients with GC and is significantly related to the overall survival prognosis of patients^[^27^]^. However, there is a lack of reports on how P2X7R affects the progress of GC cells. Therefore, we explored the role of P2X7R regulating the progression of GC and provided a new theoretical basis.

Recently, different studies have found that ATP in the tumor microenvironment has a certain effect on the activity of tumor cells^[^28-31^]^. Studies have found that platelet secretion of ATP can promote the migration and metastasis of tumor cells by acting on P2Y2 receptor, while lack of ATP reduces tumor metastasis^[^32^]^. Other studies have also found that ATP and its analogue BzATP activate the P2X7R to activate the AKT pathway to promote the migration and metastases of breast cancer cells^[^10^]^. In this study, we found that ATP and BzATP increased intracellular calcium concentration in AGS and HGC-27 cells, and promoted the proliferation of GC cells. While P2X7R antagonists A438079 and AZD9056 obtained the opposite result. The experimental results we obtained are consistent with the results of most previous studies, indicating that ATP can play a regulatory role in tumor progression by acting on P2X7R. However, the specific molecular mechanism of ATP regulating the metastasis of tumor cells needs to be explored in depth.

Extracellular ATP does not directly affect the fate of tumors, but indirectly regulates the progression of tumors by acting on some other molecular substances (such as immune cells, membrane receptors (P2X))^[^33,34^]^. The P2X7R belongs to the ATP cation channel receptor and is expressed in most tumors, such as pancreatic cancer, colorectal cancer, prostate cancer, and non-small cell lung cancer, which affects the progression of tumors^[^35-37^]^. Under physiological conditions, ATP concentration is low and P2X7R has a low affinity for ATP, which leads to restricted activation of P2X7R. However, when the body is in a pathological condition such as tumor, cells can release a large amount of ATP, resulting in a sharp increase in the concentration of extracellular ATP, which activates the P2X7R on the membrane of tumor cells, opens ion channels, and affects the tumor cell fate^[^38^]^. Activation of P2X7R by ATP or BzATP can promote EMT, migration and invasion of A549 cells, hut this effect can be attenuated by P2X7R antagonist A438079^[^39^]^. The *in vivo* results showed that P2X7R activation promoted the expression of EMT and PI3K/Akt in transplanted tumor, indicating that P2X7R promotes the migration and invasion of A549 cells by activating PI3K/Akt signal pathway^[^39^]^. P2X7R activation can enhance and change the actin cytoskeleton rearrangement of lung cancer cells, thus promoting the migration ability of lung cancer cells^[^40^]^. Moreover, some *in vivo* studies have further confirmed that P2X7R activation promotes tumor growth^[^41-43^]^. The results obtained in this study found that activation of P2X7R increased the glycogen content in GC cells, enhanced the stress of actin fibers in cells, changed the morphology of cells, and promoted the migration and invasion of GC cells. Conversely, P2X7R antagonists reversed ATP-induced cell changes. However, in the absence of ATP, P2X7R antagonist alone had no obvious inhibitory effect. This indicates that the activity of P2X7R is limited under basic conditions. When the agonist is present, P2X7R is activated to play a functional role, matching the biological functional characteristics of the P2X7R gene. Moreover, we knocked down P2X7R expression by transfecting AGS and HGC-27 cells with siRNA and obtained similar results. Additionally, *in vivo* experiments showed that ATP-induced activation of P2X7R promoted the growth of GC, while the application of AZD9056 inhibited ATP-induced tumor growth. Our current findings indicate that P2X7R has an important regulatory effect on the progression of GC.

EMT is an important feature of tumor metastasis. EMT-related gene markers such as E-cadherin, vimentin, Snail, etc., these genes play an important role in the adhesion, differentiation and migration of tumors cells^[^22,44,45^]^. ATP and P2X7R play an important role in tumor metastasis and EMT formation^[^46-48^]^. Studies have shown that knocking down the expression of P2X7R can significantly inhibit the expression of EMT/invasion-related genes Snail, Claudin-1, IL-8 and MMP-3 induced by ATP or BzATP, and inhibit the invasion, metastasis and EMT of prostate cancer cells^[^49^]^. Other studies have also shown that P2X7R activation increases the expression level of E-cadherin and MMP-9, and promotes the migration and invasion of breast cancer cells^[^10^]^. In this study, the results showed that ATP and BzATP increased the expression levels of EMT/ metastasis related genes (such as vimentin, snail and N-catenin), and decreased the expression of E-cadherin in GC cells. While P2X7R antagonists A438079 and AZD9056 inhibited the changes in the expression level of these genes induced by ATP. These data indicate that P2X7R can regulate the expression of EMT-related genes and play a role in the metastasis and EMT formation of GC cells.

The results obtained in this study indicate that activation of P2X7R promotes the proliferation, migration, and invasion of GC cells, regulates the expression of EMT-related genes, and promotes the metastasis and EMT formation of GC cells. While P2X7R antagonists or knock down the expression of P2X7R inhibit the proliferation and metastasis of GC cells induced by ATP. The possible mechanism may be that P2X7R activation is mediated by activation of P13/AKT/GSK-3beta signal pathway. Our current findings provide a new theoretical basis and data support for the role of P2X7R regulating the progression of GC, revealing the functional role and possible molecular mechanisms of P2X7R in the progression of GC. The results obtained in this study are consistent with the results obtained in previous studies of P2X7R in other tumors, revealing that the P2X7R may serve as a new potential molecular target for the prevention and treatment of GC. Therefore, inhibiting P2X7R activity or reducing P2X7R expression may become a molecular pharmacological target for GC treatment and is expected to become an important detection method for the prevention and treatment of GC in the future. However, the specific molecular mechanism by which P2X7R regulates GC progression requires more future research to explore, including using transcriptomics and proteomics.

There are some shortcomings in this study, which are also the direction we will explore and solve in the later period. For example, the concentration or dose level of the P2X7R antagonist used in this experiment. Although the dosage used can antagonize the activity of P2X7R and inhibit the growth of GC cells, whether higher doses can produce overlapping inhibitory effects or bring negative effects, and which dosage can achieve the best pharmacological effects on inhibiting the growth and metastasis of GC cells, require more time and effort to explore in the later stage. Moreover, it is understood that immune escape and immunosuppression in the tumor microenvironment are key factors in tumor growth and metastasis^[^50,51^]^. The P2X7R can be expressed in these immune cells in the tumor microenvironment, thereby regulating the activity of these immune cells and participating in tumor progression^[^52,53^]^. However, whether P2X7R affects the activity of these immune cells and thus affects the progression of GC is also a flaw in this study. Therefore, in the later stage, we will focus on studying how the P2X7R regulates the activity of immune cells and thus regulates the growth and metastasis of GC cells, so as to better understand the more detailed molecular basis of the P2X7R involvement in GC progression. Furthermore, although these data support our results and conclusions, the limited sample size used in this study may have a certain impact on the results obtained. Therefore, more sample sizes and data are needed to better support the conclusions obtained. In addition, there may also be certain shortcomings in the construction of animal models. Based on most previous studies, we established a subcutaneous tumor-forming animal model in nude mice by subcutaneous injection of GC cells^[^39,54,55^]^. Subsequently, P2X7R antagonists were used to inhibit tumor growth, without using knockdown P2X7R cell lines or transgenic animal models to further study. Therefore, in later research, we will further focus on exploring the influence of P2X7R on the changes in the tumor microenvironment of GC *in vivo* by establishing a transgenic mouse animal model, and understand the possible molecular mechanisms of P2X7R regulating the changes in the tumor microenvironment and mediating the growth and metastasis of GC from different perspectives. In short, the data obtained in this study reveal that the P2X7R may be used as another potential molecular target for the prevention and treatment of GC. However, before the P2X7R can be used as a molecular target for the prevention and treatment of GC in clinical patients, more data and practice are needed in the future to verify.

## Data Availability

All data generated or analyzed during this study are included in this article. And we have not used other data that has already been published. All the data presented in this article are original results derived from this study.

## Research Registration Unique Identifying Number (UIN)

This study is in vitro and includes some in vivo studies, and animal ethics has been agreed.
